# Transcriptional programs are activated and microRNAs are repressed within minutes after mating in the *Drosophila melanogaster* female reproductive tract

**DOI:** 10.1186/s12864-023-09397-z

**Published:** 2023-06-27

**Authors:** Sofie Y. N. Delbare, Asha M. Jain, Andrew G. Clark, Mariana F. Wolfner

**Affiliations:** Department of Molecular Biology & Genetics, Cornell University, Ithaca, NY 14853 USA

**Keywords:** *Drosophila melanogaster*, Post-mating response, Transcriptome, microRNAs, Reproductive tract, Exosomes

## Abstract

**Background:**

The female reproductive tract is exposed directly to the male’s ejaculate, making it a hotspot for mating-induced responses. In *Drosophila melanogaster*, changes in the reproductive tract are essential to optimize fertility. Many changes occur within minutes after mating, but such early timepoints are absent from published RNA-seq studies. We measured transcript abundances using RNA-seq and microRNA-seq of reproductive tracts of unmated and mated females collected at 10–15 min post-mating. We further investigated whether early transcriptome changes in the female reproductive tract are influenced by inhibiting BMPs in secondary cells, a condition that depletes exosomes from the male’s ejaculate.

**Results:**

We identified 327 differentially expressed genes. These were mostly upregulated post-mating and have roles in tissue morphogenesis, wound healing, and metabolism. Differentially abundant microRNAs were mostly downregulated post-mating. We identified 130 predicted targets of these microRNAs among the differentially expressed genes. We saw no detectable effect of BMP inhibition in secondary cells on transcript levels in the female reproductive tract.

**Conclusions:**

Our results indicate that mating induces early changes in the female reproductive tract primarily through upregulation of target genes, rather than repression. The upregulation of certain target genes might be mediated by the mating-induced downregulation of microRNAs. Male-derived exosomes and other BMP-dependent products were not uniquely essential for this process. Differentially expressed genes and microRNAs provide candidates that can be further examined for their participation in the earliest alterations of the reproductive tract microenvironment.

**Supplementary Information:**

The online version contains supplementary material available at 10.1186/s12864-023-09397-z.

## Background

Across species, the female reproductive tract undergoes significant changes post-mating to create an optimal microenvironment for fertilization. In mammals, the male’s ejaculate activates the immune system in the uterus, and this facilitates conception and embryo implantation [1]. In insect vectors of disease, mating regulates multiple functional classes of genes in the female reproductive tract, including genes involved in the immune response, proteolysis, and genes with neuronal functions [2–5], but exactly which of these genes are key regulators of reproductive success is not known.

In *Drosophila melanogaster*, the female lower reproductive tract consists of the genital chamber (which includes uterus, vagina and vulva), female accessory glands, and two types of sperm storage organs, the seminal receptacle (the primary sperm storage organ) and paired spermatheca (longer-term sperm storage) [6–8]. The upper reproductive tract consists of one common and two lateral oviducts [6, 7, 9]. Both upper and lower reproductive tracts are extensively innervated [9–14].

Tissues within the female reproductive tract undergo marked changes in response to mating at the level of gene expression, protein abundance and localization, and whole tissue morphology. In *D. melanogaster*, a mating lasts on average 20 min. Within the first 10 min of mating, sperm, seminal fluid proteins, and other ejaculate components such as microcarriers (lipid-containing structures that bind some seminal proteins) and exosomes, are transferred, and a mating plug is formed to prevent the ejaculate from leaking out of the tract until sperm storage is complete [6, 15–19]. Microcarriers disassemble in the uterus within 30 min after the start of mating [18], and sperm storage starts already before copulation ends. Sperm storage is typically fully completed by 5–6 h post-mating [6, 8, 20], while the mating plug is ejected on average 3 h after mating [21].

During copulation, the morphology of the uterus starts to change. The uterus, which is S-shaped and contracted in unmated females, becomes round and turgid by 35 min after the start of mating [22–24]. Concurrently, changes occur in the release of vesicles and neuromodulators from neurons that innervate the reproductive tract, and a few hours after mating neuron bouton numbers have increased [9, 11, 12, 25–27]. By 90 min after the start of mating, the oviduct, which is coiled in unmated females, straightens out [23]. These morphological changes are accompanied by changes in myofibril organization and cellular adhesion [10, 11, 27]. Alterations in RNA and protein levels have mostly been measured at 3 h post-mating and later. The RNAs involved affect functions related to transcription, translation, proteolysis, the cytoskeleton, immune response, signal transduction, and neuron and muscle function [28–32]. The aggregate of these changes is needed to allow successful sperm storage and ovulation.

Even though many key events rapidly take place in the female reproductive tract post-mating, there is a lack of transcriptome resources that characterize this tissue at very early timepoints. An exception is Mack et al. (2006) [29], where microarrays were used to measure transcript levels in the female reproductive tract at 0 h after mating. However, 0 h post-mating does not indicate a well-defined, precise time point. Delbare et al. (2023) [33] demonstrated, using a fine-grained time series of the female head’s transcriptional response to mating, the importance of sampling well-defined and early timepoints to capture the earliest regulators and transient dynamics.

In addition to characterizing mating-induced changes in the female reproductive tract, there is also an interest in understanding which female and male molecules regulate these changes.

There is evidence that microRNAs contribute to the regulation of reproductive processes. In mammals, microRNAs carried in male exosomes that fuse with sperm influence embryogenesis [34]. In *D. melanogaster*, certain microRNAs regulate post-mating responses [35–37], and the abundance of many microRNAs is altered after mating [38], with distinct responses in the abdomen vs. the head and thorax, as measured at 3 h post-mating [39]. MicroRNAs have been hypothesized to be important regulators that can reprogram gene expression in the female reproductive tract [40], but genome-wide microRNA expression changes specific to the *D. melanogaster* female reproductive tract have not been reported, at any timepoint, and there is no functional evidence that microRNAs are used to regulate the mRNA abundance of genes involved in reproductive tract-specific changes post-mating.

On the male side, previous work has shown that specific *D. melanogaster* male seminal proteins induce morphological changes in the female reproductive tract and are needed for sperm storage [23, 24, 41–43], and that transcriptome changes in whole females at 1–3 h after mating are influenced by copulation itself, by sperm, and by seminal fluid proteins [44]. Specifically, McGraw et al. (2004) studied the effects of seminal fluid proteins on the female’s transcriptome by ablating male accessory gland main cells, which are predominant producers of seminal fluid proteins [45, 46]. However, male accessory glands also contain ~ 40 secondary cells, which are larger than the main cells and are filled with vacuoles [47, 48]. Secondary cells contribute to the composition of seminal fluid in at least two ways. First, secondary cells produce, and influence, the abundance of certain seminal fluid proteins in the ejaculate [49–53]. Second, secondary cells are the source of exosomes, membrane-bound vesicles with a diameter of 40–100 nm. Exosomes carry RNAs and proteins, and are involved in intercellular communication [54]. In mammals, exosomes derived from the prostate fuse with sperm and affect sperm function [54]. In *D. melanogaster*, exosomes that are transferred to the female during mating associate with the female reproductive tract lining and fuse with sperm within 30 min after the start of mating [19]. Males that do not transfer secondary cell-derived exosomes (and also have some other abnormalities in their seminal plasma) due to inhibition of BMP signaling in secondary cells fail to induce refractory mating behavior in their mates, without altering egg laying, and the males’ sperm competitive abilities are altered [19, 53]. It is unknown what active molecules are held within these exosomes, and whether male exosomes contribute to the regulation of the female reproductive tract transcriptome.

To obtain a comprehensive view of transcriptional changes in the female reproductive tract early after mating, we sequenced RNAs and microRNAs of the reproductive tracts of unmated females and females collected at 10–15 min after the end of mating. These females were mated either to a control male or a male that cannot transfer exosomes, to assess the effect of exosomes on the early female reproductive tract transcriptome. Regardless of the male’s genotype, we observed mating-induced changes in the expression of genes and of microRNAs, that have putative targets among the differentially expressed genes, and which likely contribute to a switch from an unmated to a mated reproductive tract.

## Results

### The female reproductive tract undergoes small-magnitude changes in transcript abundance within 15 min after mating, including with males that have inhibited BMP signaling in their secondary cells

**Fig. 1:**
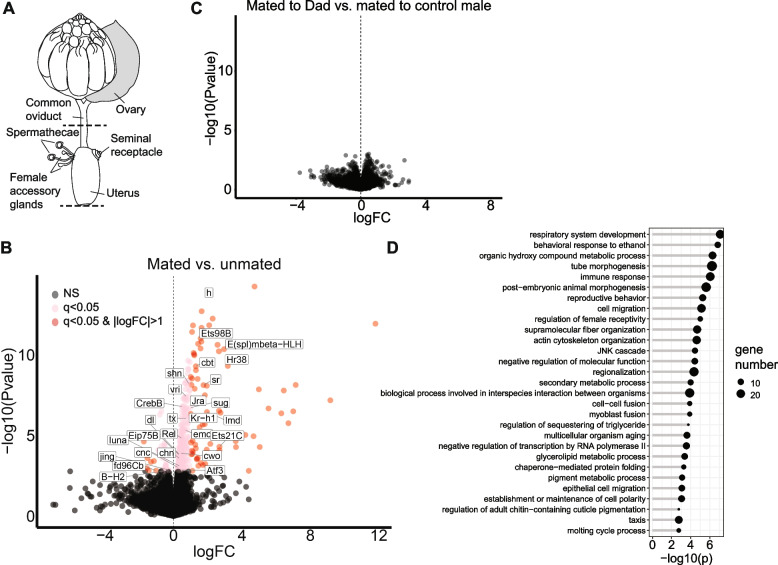
Mating induces differential abundance of RNAs in the female reproductive tract. **A** Schematic of the female reproductive tract, showing sampled tissues in between dashed lines. Image adapted from Mack et al. (2006) [29]. **B** Volcano plot showing genes that are differentially expressed between unmated females and females mated to *w*^*1118*^ males. Differentially expressed genes that encode transcription factors are annotated with gene symbols. **C** Volcano plot showing absence of differential expression between females mated to Dad vs control males. *NS* Not significant, *q* = *q-value*; logFC = log_2_ fold change; dark red dots: *q-value* < 0.05 and ≥ twofold change in expression; positive fold changes indicate an upregulation post-mating, negative fold changes indicate a downregulation post-mating. **D** GO term enrichment analysis for “Biological Process”. All GO terms shown have a *q-value* < 0.05

To investigate transcriptome changes in the female reproductive tract, we dissected the entire lower reproductive tract and the lower half of the common oviduct for RNA sequencing (Fig. 1A; on average 64 reproductive tracts per sample). We did not remove the fat body tissue that surrounds the spermathecae. A pairwise comparison between unmated females and females mated to *w*^*1118*^ males detected 327 genes that were differentially expressed at 10–15 min after the end of mating (*q-value* < 0.05; based on 3 biological replicates per treatment; Fig. 1B; Table S1). Of these 327 genes, the majority were upregulated in mated vs. unmated females (294 up; 33 down), and 82 genes underwent at least a twofold change in expression in mated vs. unmated females (74 up; 8 down).

We next studied the effects on the female reproductive tract transcriptome of mating with “Dad” males, who cannot transfer exosomes when overexpression of the BMP-inhibitor Dad is induced at 29°C [19, 53, 55]. (We note that since flies are ectotherms, 10 to 15 min after mating at 29°C might not perfectly match up with 10 to 15 min after mating at 21°C.) A pairwise comparison between females mated to control males and females mated to Dad males (both collected at 29°C) identified 0 differentially expressed genes, even when using a *q-value* cutoff of 0.1 (based on 3 biological replicates per treatment; Fig. 1C; Table S2). To ensure that this result was not caused by problems with the transgenic constructs, we compared the transcriptomes of Dad and control males. This analysis detected a significant upregulation of the *Dad* gene in Dad vs. control males (log_2_ fold change = 1.98, *q-value* = 0.014; see Additional Results), indicating that the overexpression construct worked. We further conducted a receptivity assay on the mates of Dad males. Mates of Dad males have been reported to be more receptive to remating compared to mates of control males [19], and this is indeed what we observed in our experiment (Fisher’s exact test *p-value* = 1.3*10^–5^; see Additional Results and Fig. S1).

The predominant upregulation of genes in mated vs. unmated females suggests that the female reproductive tract responds to mating by activating regulatory programs that turn the tract into a “mated” state, rather than downregulating regulatory programs that define an “unmated” tract. Our results further suggest that BMP dependent components of secondary cells, such as exosomes, are not needed for these early reproductive tract-specific changes, at least based on bulk RNA-seq.

### Distinct functional classes of genes are regulated in the reproductive tract within 15 min after mating

To better understand the biological relevance of these transcriptome changes, we employed GO term enrichment analysis. Since previous studies of the female reproductive tract transcriptome (e.g. [32]) identified an overrepresentation of genes that encode secreted proteins, we first ran a GO term enrichment analysis for “Cellular Component (CC)”. We indeed found a significant enrichment of genes associated with the term “extracellular space”. Other significantly enriched “CC” GO terms include “lipid droplet”, “subsynaptic reticulum” and “cell cortex”.

Using a GO term enrichment analysis for “Biological Process”, we identified 210 significantly enriched GO terms (Table S3), which were summarized in 29 GO terms using the R package rrvgo [56] (Fig. 1D). Below we discuss a subset of representative GO terms. First, we identified 21 genes linked with the GO term “immune response”. Among these genes are ones that encode signaling molecules such as *cactus, Relish* and *dorsal.* Notably, antimicrobial peptides, immune effector molecules that are upregulated after mating at later timepoints [28, 32, 39, 44, 57–59], were not among our differentially expressed gene set. Related significantly enriched GO terms included “wound healing” and “melanization defense response”. These include genes involved in the clotting reaction and might reflect the female’s response to copulatory wounding [23, 60]. Second, we detected the GO terms “cytoskeleton organization”, “cell migration”, “tissue morphogenesis” (including “neuron projection morphogenesis”), and “myoblast fusion”. These terms might relate to processes that help heal the reproductive tract after copulatory wounding, but they might also relate to the morphological changes the reproductive tract undergoes, and remodeling of the tract lining, musculature and/or innervation, in response to mating. Third, we identified 17 genes involved in “cellular lipid metabolic process”, suggesting a change in energy metabolism post-mating. Fourth, we identified several genes associated with the GO term “reproductive behavior”. Among these genes was *jhamt*, which is involved in the regulation of Juvenile Hormone, a hormone that is upregulated after mating [61, 62]. We also detected RNAs of 9 genes that encode male seminal fluid proteins [46] (*Acp95EF, Acp36DE, Mst57Da, Mst57Db, Acp53C14b, Dup99B, Sfp96F, Anp* and *Acp98AB*). Genes that encode seminal fluid proteins are primarily expressed in males [46, 63–65], but there is evidence that some can also be expressed in females [32, 66, 67]. We were curious whether the RNAs that we detected from genes that encode seminal fluid proteins originated from the female, or whether they had been transferred by the male. Since we had RNA-seq samples of males, virgin and mated females, and since the females and males in our mating experiment had different genotypes, we visually inspected reads aligning to these genes using the Integrative Genomics Viewer [68]. For two genes (*Anp, Acp53C14b*), reads were present in males that spanned these genes’ introns, suggesting the presence of unspliced pre-mRNA. However, in mated females, no reads were found in the introns of *Anp* or *Acp53C14b*, indicating that only mature spliced mRNA was detected in females. It is possible that these mature transcripts were transferred by the male. In addition, for *Acp53C14b*, we identified SNPs that were shared among reads of males and mated females, and that differed from SNPs in reads found in unmated females, again suggesting that transcripts may have been transferred from the male to the female during mating (Fig. S2). As can be seen in Fig. S2, a small number of reads align to *Anp* and *Acp53C14b* in unmated females as well, suggesting that in addition to potential transfer, some low-level expression occurs in females as well. For other genes encoding seminal fluid proteins, no unambiguous SNP genotype information was available to indicate male vs. female origin.

Finally, a GO term enrichment analysis for “Molecular Function” identified a significant enrichment of genes that encode transcription factors (27 genes, *q-value* = 0.03). All except 3 of these were upregulated in response to mating (Fig. 1B).

In addition to protein-coding genes, mating also regulated the abundance of 12 long noncoding RNAs and 4 antisense RNAs. Of these noncoding RNAs, 3 are microRNA hosts.

Taken together, we identified specific classes of genes that respond to mating in the female’s reproductive tract and that likely contribute to optimizing the tract environment for fertilization. The enrichment of upregulated transcription factors further supports our hypothesis that mating activates regulatory programs to prepare the tract for fertilization events, rather than simply suppressing virgin-specific gene networks.

### Rapid post-mating transcriptome changes likely occur in multiple tissues across the female reproductive tract

**Fig. 2:**
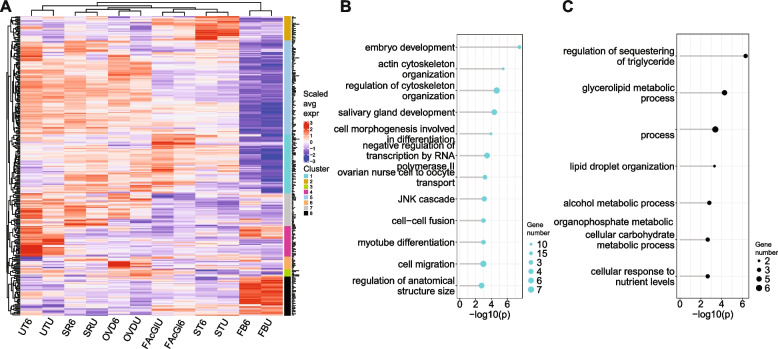
Rapid post-mating transcriptome changes likely occur across multiple tissues within the female reproductive tract. **A** Heatmap showing average expression values from McDonough-Goldstein et al. (2021) [32] across reproductive tract tissues, in unmated females (U) and at 6 h after mating [6], for differentially expressed genes in our dataset. UT = uterus; SR = seminal receptacle; OVD = oviduct; FAcGl = female accessory gland; ST = spermathecae; FB = fat body. **B** GO term enrichment analysis for “Biological Process” for genes in cluster 1. **C** GO term enrichment analysis for “Biological Process” for genes in cluster 8. All GO terms shown have a *q-value* < 0.05

McDonough-Goldstein et al. (2021) [32] report an atlas of gene expression changes at 6 and 24 h post-mating in different tissues within the female reproductive tract (the oviduct, uterus, female accessory glands, spermathecae, seminal receptacle, and the fat body surrounding the tract). We made use of this resource to investigate whether any of our differentially expressed genes have an expression bias to any specific tissue(s) within the reproductive tract. This could help to establish whether all tissues across the tract, or only specific tissues, are involved in this rapid (10–15 min post-mating) transcriptomic response. For the genes that were differentially expressed in our study, we retrieved their average expression in reproductive tract tissues, in unmated females and at 6 h after mating, as calculated by McDonough-Goldstein et al. (2021) [32] (Table S4). Using hierarchical clustering of these average expression values, we obtained 8 clusters of genes, and found significant GO term enrichment for two clusters (Fig. 2A; Table S4).

Cluster 1 was enriched for genes with functions in cytoskeleton organization, cell morphogenesis, migration, and differentiation (Fig. 2B). The genes in cluster 1 have high average expression in the oviduct, sperm storage organs and female accessory gland. Cluster 8 was enriched for genes that have roles in lipid and carbohydrate metabolism (Fig. 2C), such as *Dawdle, brummer, Lipid storage droplet-1, Lipid storage droplet-2* and the transcription factor *sugarbabe*. Based on the dataset from McDonough-Goldstein et al. (2021) [32], these genes tend to be expressed predominantly in the fat body that surrounds the reproductive tract. Cluster 2 was not enriched for any functions, but expression of genes in this cluster tends to be spermathecae-specific and this cluster includes the known spermathecal gene *Send2* [69]. Another gene of interest in this cluster is *bond*, which is also expressed in the male ejaculatory bulb, where it is required for synthesis of cis-vaccenyl acetate, one of the pheromones transferred by the male during mating [70]. Integration with the reproductive tract expression atlas from McDonough-Goldstein et al. (2021) [32] suggests that mating has effects on the transcriptome of multiple tissues across the female reproductive tract, already within 15 min post-mating.

### MicroRNAs’ abundances change in the female reproductive tract in response to mating

**Fig. 3:**
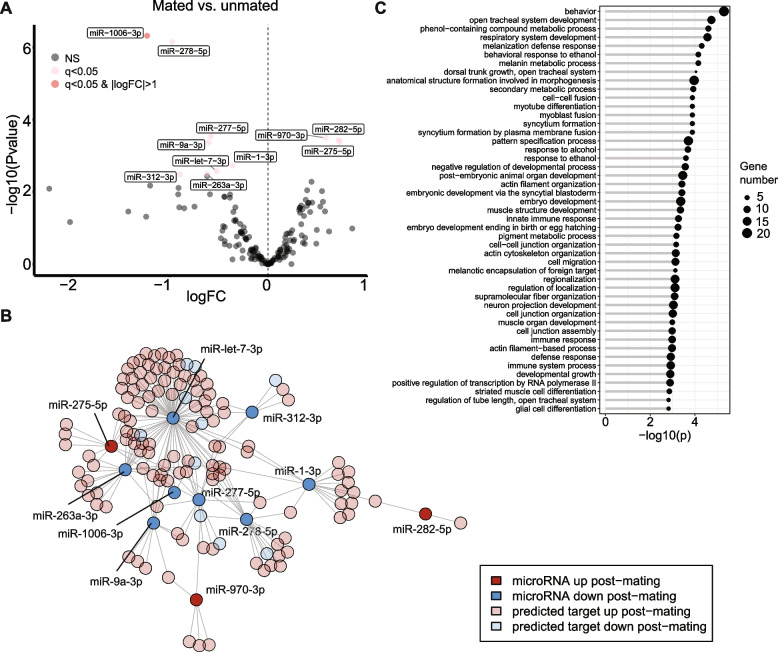
MicroRNAs respond to mating in the female reproductive tract. **A** Volcano plot showing microRNAs that are differentially abundant after mating. NS = not significant; q = *q-value*; logFC = log_2_ fold change; dark red dots: *q-value* < 0.05 and ≥ twofold change in expression; light red dots: *q-value* < 0.05; positive fold changes indicate an upregulation post-mating, negative fold changes indicate a downregulation post-mating. **B** Network showing edges between microRNAs and predicted targets from the differentially expressed mRNA-seq gene set. **C** GO term enrichment analysis for “Biological Process” for predicted targets of differentially abundant microRNAs. All GO terms shown have a *q-value* < 0.05

We detected a total of 164 expressed microRNAs across mated and unmated reproductive tract samples, after filtering out microRNAs with very low expression (see Methods). Of these microRNAs, 11 were differentially abundant after mating (*q-value* < 0.05; based on 3 biological replicates for unmated females and 2 biological replicates for females mated to *w*^*1118*^ males; Fig. 3A; Table S5). Only one microRNA, *mir-1006-3p*, had a larger than twofold change in expression. A second microRNA, *mir-278-5p*, narrowly missed the twofold expression cutoff (log_2_ fold change = -0.97). As for the mRNA-seq dataset, mating with Dad males had no significant effect on the mated female’s microRNA transcriptome (based on 2 biological replicates for females mated to control males and 3 biological replicates for females mated to Dad males; Table S6). The absence of differences between females mated to control vs. Dad males also suggests that BMP-dependent secondary cell products, such as secreted exosomes, do not transfer microRNAs to females in amounts that we can detect with a bulk RNA-seq approach.

Of the 11 differentially abundant microRNAs, 8 were downregulated. Thus, the overall direction in which microRNA abundances change is the opposite of what we observe for the mRNA-seq dataset, in which most genes were upregulated in response to mating. To understand possible biological roles of these microRNAs, we retrieved their putative targets from TargetScan [71]. Of the set of 327 differentially expressed genes, 130 were predicted targets of differentially expressed microRNAs (Fig. 3B). Of these 130 predicted microRNA targets, 120 were upregulated after mating. This may be consistent with a derepression of target genes whose expression was attenuated by those microRNAs in unmated females.

A GO term enrichment analysis of predicted targets showed enrichment of terms related to the immune- and wound healing response, muscle development, cytoskeleton- and cell junction organization (Fig. 3C; Table S7). We further investigated predicted targets of the two most significantly differentially expressed microRNAs. For *mir-1006-3p*, 3 predicted targets were among the differentially expressed mRNA-seq gene set (*aay, luna, krz*). Of interest is *kurtz* (*krz*), which stimulates the internalization of G protein-coupled receptors to downregulate their activity [72], since a key receptor for the seminal fluid protein Sex Peptide, the Sex Peptide Receptor, is a G protein-coupled receptor [73]. For *mir-278-5p*, 18 predicted targets were part of the differentially expressed mRNA-seq gene set and were enriched for “anatomical structure morphogenesis”.

As written above, 3 differentially expressed long noncoding RNAs are microRNA hosts, and we found that 2 of these genes host microRNAs that are also differentially expressed in our dataset. *lncRNA:CR45916* hosts *mir-9a*, and *lncRNA:CR43314* hosts *mir-263* (also called *mir-bft*). Interestingly, both microRNAs are downregulated in mated females, while their host lncRNAs are upregulated, suggesting complex regulatory mechanisms potentially involving post-transcriptional regulation.

Our data suggest that microRNAs might be used in the unmated female reproductive tract to repress genes that are needed to remodel the tract after mating. Signals from mating might then lead to a derepression of these genes via the downregulation of microRNAs.

## Discussion

During and shortly after mating, the female reproductive tract undergoes tissue-level changes that are essential for reproductive success. While immediate changes are likely driven by proteins that are already present in the reproductive tract, we show that even at a very early timepoint post-mating, the reproductive tract transcriptome has already undergone marked changes.

The female reproductive tract has been suggested to be “poised” to respond to mating, based on observations that gene expression changes at early timepoints are of only small magnitude [29, 44], and based on the presence of vesicles with neuromodulators that are at the ready in neurons that innervate the reproductive tract of unmated females [9]. Our results reveal another way in which the female reproductive tract is poised to respond to mating, since (i) most of our differentially expressed genes underwent small magnitude fold changes (although it is possible that these genes’ fold changes will still increase in magnitude after the 15 min timepoint that we sampled); (ii) mating rapidly upregulates RNA abundance, including of genes that encode transcription factors, which might activate transcriptional programs needed for tract remodeling; (iii) mating rapidly represses microRNAs, which likely leads to the derepression of genes needed for tract remodeling.

Below, we discuss our results within the context of previously published studies that report mating-induced transcriptome changes and discuss the function of differentially abundant transcripts in light of known tissue-level changes that occur in the reproductive tract.

### Very early transcriptome changes in the reproductive tract might be transient

We compared our results with a published dataset that is most similar to ours in terms of tissue and timepoint sampled. Mack et al. (2006) identified 71 differentially expressed genes at 0 h post-mating, using microarrays of the female reproductive tract [29]. None of these 71 genes overlap with our set of differentially expressed genes, but they are enriched for the same GO terms, such as “regulation of nervous system development”, “anatomical structure morphogenesis”, “cell junction organization” and “regulation of metabolic process”.

Comparing our set of 327 genes with a recent RNA-seq study of the female reproductive tract of the wildtype strain LH_M_ at 6 and 24 h after mating yields 69 genes in common at 6 h (no GO term enrichment) and 50 genes in common at 24 h (enriched GO terms relate to proteolysis and the immune response) [32]. Overall, at 6 and 24 h after mating, McDonough-Goldstein et al. (2021) [32] highlight GO terms related to transcription, translation, the immune response and proteolysis, but terms related to e.g. tissue morphogenesis or cytoskeleton organization are notably absent. Differences between our dataset (10–15 min post-mating) and the McDonough-Goldstein et al. (2021) [32] dataset (6 and 24 h post-mating) could be due to differences in strains or experimental setup, but are likely at least in part driven by the timepoints at which samples were collected. We suggest that many of the early gene expression changes might be transient. Several hours post-mating, full remodeling of the reproductive tract has mostly been achieved [6, 8, 9, 12, 20, 22, 23], and the transcriptional programs that induced these changes may have gotten shut down. McDonough-Goldstein et al. (2021) [32] also found that most gene expression changes in the reproductive tract were transient, with many changes at 6 h post-mating no longer detectable at 24 h. Other mating-induced effects are transient as well, such as those of transferred pheromones, and certain post-mating transcriptome changes in the *D. melanogaster* female head [33, 74, 75].

MicroRNAs have received less attention than mRNAs in studies of the post-mating response. Global changes in microRNA expression post-mating have been reported in whole females (after multiple matings taking place over several days) [38] and in the head/thorax and abdomen separately (at 3 h post-mating) [39]. None of the differentially abundant microRNAs in our dataset are differentially regulated post-mating in [38, 39]. Again, differences in sampled tissues or experimental setup might explain the lack of overlap. Alternatively, differential regulation of these microRNAs might only be required very early on in the post-mating response to achieve reproductive tract remodeling but does not need to be sustained long-term. More so, the transient regulation of microRNAs might be key to the transient modulation of mRNA targets.

Three studies investigated functional roles of microRNAs in the female post-mating response [35–37]. Garaulet et al. (2020, 2021) [35, 36] focused on neurons, while Fricke et al. (2014) [37] focused on whole fly expression. Of the microRNAs they studied, only *mir-278*, studied by Fricke et al. (2014) [37], was also differentially expressed in our reproductive tract dataset. However, *mir-278* changes in a different direction; down after mating in our dataset, and up after mating in Fricke et al. (2014) [37]. Since Fricke et al. (2014) [37] sampled at 3 h after mating, the difference in timepoint, or the difference in sampled tissue, could explain the difference in fold change direction. It also suggests that *mir-278* might play a role in diverse processes in the response to mating.

Overall, comparing our results with published studies suggests that gene programs that rapidly respond to mating in the reproductive tract might be triggered only transiently, and might be shut down again once remodeling has been achieved. This potential transient nature highlights the importance of studying samples at early timepoints that match the timing of phenotypic events induced by mating.

### Transcriptomics analysis identifies candidate genes with distinct roles in the female reproductive tract’s response to mating

Even though most RNAs underwent small-magnitude fold changes, differentially expressed genes were enriched for specific functional classes, and they might occur in specific tissues within the reproductive tract.

First, we observed increases in the transcript levels of genes that mediate wound healing. Copulatory wounding is known to occur in *Drosophila* [23, 60]. In addition, it has been observed that several seminal fluid proteins enter the female’s hemolymph during copulation [50, 76, 77] and at least two seminal fluid proteins act on the female’s central nervous system [73, 78, 79], indicating that they have entered the female’s circulation. While it has been suggested that seminal fluid proteins can traverse the posterior vagina because of its thinner lining [77], wounding would provide easy access to the female’s hemolymph. While this could be advantageous to the male, for females, wounding could result in a cost, for example if pathogens can enter the hemolymph via the wound. Our data indicate that the female rapidly responds to this potential cost by upregulating mRNAs of genes important for wound healing. The post-mating upregulation of transcripts involved in tissue morphogenesis and cytoskeleton organization could reflect a general reorganization of reproductive tract musculature and epithelia to accommodate the conformational changes in the tract, needed for sperm storage and ovulation [11, 23]. Using microscopy and immunohistochemistry, Kapelnikov et al. (2008) [27] observed that mating induced an increase in the number of myofibrils, and altered cellular junctions in the epithelium of the oviduct. Our transcriptome data, and integration with the dataset from McDonough-Goldstein et al. (2021) [32], suggest that similar changes occur in the female accessory gland and sperm storage organs as well, in addition to the oviduct (cluster 1). Our data further indicate, based on GO term enrichment analysis of putative microRNA targets, that these changes might be regulated by microRNAs.

By 20 min after the start of mating, neuromodulator dynamics and bouton number change in the neurons that innervate the reproductive tract [9, 11, 12, 25, 27]. Differentially expressed genes (including predicted microRNA targets) associated with the GO term “neuron projection development” might play a role in these changes, strengthening the interaction between the nervous system and reproductive tract muscles in a mated female.

We further observed changes in metabolic genes, which, according to the dataset from McDonough-Goldstein et al. (2021) [32], might occur in the fat body that surrounds the reproductive tract. We hypothesize that these metabolic changes are needed to supply energy to reproductive tract tissues to make the remodeling of the tissue possible. These metabolic changes are interesting because they are lipid/carbohydrate related, and not protein related, even though we know that protein metabolism is upregulated at later timepoints after mating, likely to sustain egg production [39, 80, 81]. Thus, energy requirements might differ locally and in a time-dependent fashion in the female’s post-mating response. Alternatively, upregulation of lipases in the reproductive tract shortly after mating could contribute to the breakdown of lipid microcarriers in the ejaculate, which happens within 30 min after the start of mating [18].

We also observed changes in immune gene transcript levels. Whereas transcripts of immune response genes and specifically antimicrobial peptides (AMPs) are upregulated at several hours post-mating in whole bodies [28, 44, 57, 58, 82–86], we did not observe changes in AMP transcripts at 10–15 min after the end of mating in the reproductive tract. However, we do observe an upregulation of RNAs of components of the Toll and Imd pathways that function upstream of AMP transcription (e.g. *Relish, cactus*). This upregulation of immune transcripts implies that the reproductive tissue prepares itself for increased infections or prolonged exposure to unfamiliar male proteins [44, 83, 87].

Long noncoding RNAs, RNAs of at least 200 nucleotides and with no clear open reading frame, have not been thoroughly investigated for their response to mating in *D. melanogaster* females. We identified 16 lncRNAs that are differentially regulated by mating, of which 2 host microRNAs that are differentially expressed in our dataset. Long noncoding RNAs can contribute to gene regulation on epigenetic, transcriptional and post-transcriptional levels [88], and have been implicated in male reproductive biology in *D. melanogaster* [49, 89]. The long noncoding RNAs we identified are interesting candidates, in addition to the protein-coding genes and microRNAs, to further study in order to understand the regulatory mechanisms underlying post-mating reproductive tract modifications.

### *Drosophila melanogaster* males potentially transfer RNAs to females during mating

Among the upregulated RNAs in our dataset, we detected ones that are annotated as encoding seminal fluid proteins [46]. McDonough-Goldstein et al. (2021) [32] also detected RNAs of genes that encode seminal fluid proteins in the female accessory glands at 6 h post-mating. Of these, only *Acp53C14b* was also upregulated in females in our study (which focused on an earlier timepoint). Our data suggest that in addition to female expression of genes that encode seminal fluid proteins, RNAs of these genes might also be transferred to females by the male, both in the presence and absence of exosomes. Transcriptome studies performed at 0 h after mating in *Aedes* mosquitoes [2] and at 15 min after heterospecific matings between *D. arizonae* and *D. mojavensis* [90] also observed that male RNAs were transferred to the female during mating, although none of the male-derived transcripts we identified are homologous to transferred seminal fluid protein transcripts identified by [90]. In mice, microRNAs are a functional part of the male ejaculate [34, 91], and it has been hypothesized that similar mechanisms exist in *D. melanogaster* as well [92]. Our observations, in addition to those of [2, 34, 90–92] motivate the use of *D. melanogaster* to investigate how male–female crosstalk of transcripts (mRNAs as well as microRNAs) influences reproductive success.

### Lack of BMP signaling in males’ secondary cells, and thus likely the transfer of male-derived exosomes, had no detectable effect on early transcriptome changes in the female reproductive tract

Blocking BMP signaling in secondary cells has been reported to have several effects on those cells, including preventing the secretion of their exosomesinto the ejaculate [19, 53] and changes in the abundance of certain seminal fluid proteins in the male accessory gland [53]. BMP signaling in secondary cells is further required for the replenishment of dense core granules, whose role in reproduction has not yet been examined [93]. We found that mating with Dad males, whose secondary cell BMP signaling is inhibited, did not cause significant differences in female post-mating transcript abundance relative to that seen after mating with control males. This is consistent with the idea that exosome contents do not cause changes in the female’s transcriptome, although it cannot speak to effects of those contents (or other BMP-dependent molecules) at the protein level, or to transcriptome effects after 15 min post-mating.

## Conclusions

This work has revealed very early transcriptional responses to mating in the female reproductive tract. These responses encompass a predominant upregulation of protein-coding genes and a repression of microRNAs, to prepare the tract for sperm storage and fertilization events, and to heal wounds caused by copulation. We identified transcription factors and microRNAs that could be crucial regulators of these responses and that are candidate genes to further investigate for their role in early reproductive tract remodeling.

## Methods

### Fly husbandry

All females used were from the wildtype line *Oregon-R-P2*. Males were either from the line *w*^*1118*^ or were offspring with wildtype phenotype from *w*^*1118*^ males crossed to *w;esg-GAL4 tub-GAL80ts UAS-FLP/CyO;UAS-GFP nls, act* > *CD2* > *GAL4/TM6* (*esg*^*ts*^* F/O*) females (genetic controls for “Dad” males) [55]. Males with inhibited BMP signaling in the secondary cells (“Dad” males) were generated by crossing *esg*^*ts*^* F/O* females to *w;P[w* + *UAS-Dad]* (*UAS-Dad*) males (Dad acts as a BMP inhibitor) [55, 93, 94]. The latter two lines were kindly given to us by Dr. Clive Wilson. Stocks were maintained at 21 °C or 29 °C on yeast/ glucose food in a 12 h light/dark cycle.

### RNA-seq sample collection

To investigate early transcriptome changes induced by mating, we mated 3–5 day old virgin *Oregon-R-P2* females to 5 day old virgin *w*^*1118*^ males at 21 °C. Matings were observed, females were flash frozen at 10–15 min after the end of mating and virgin females were collected in parallel.

To investigate if female gene expression is impacted by the transfer of exosomes, we crossed either *w*^*1118*^ or *UAS-Dad* males to *esg*^*ts*^* F/O* virgin females at 21 °C. From the offspring of this cross, we selected male pupae once sex combs were visible, just before eclosion. Male pupae were placed in groups of 10–15 at 29 °C. The initial cross was set up at room temperature to avoid inhibition of BMP signaling during development. Transferring late-stage pupae to 29 °C will initiate the inhibition of BMP signaling in newly eclosed adult males.

Other protocols to generate Dad males allow males to eclose at 18–20 °C before shifting them to 29 °C. These males are then triple-mated before conducting any experiments to empty the accessory gland lumen of any exosomes that might have been secreted before overexpression of Dad at 29 °C [19, 53]. We did not triple-mate our males because Leiblich et al. (2012) [55] reported that previously mated males can undergo delamination of whole secondary cells during subsequent matings. This delamination of secondary cells was never observed in virgin males [55]. Because we were interested in detecting potential RNA transfer via exosomes, not whole secondary cells, we used virgin males instead of triple-mated males for the matings for our RNA-seq assays.

The afternoon before matings were set up, 2–4 day old *Oregon-R-P2* [95] virgin females were also placed at 29 °C. The next morning, 5 day old virgin males were mated to the 3–5 day old *Oregon-R-P2* virgin females at 29 °C. Matings were observed and females were flash frozen at 10–15 min after the end of copulation. Virgin females kept at 29 °C were collected in parallel. During this sample collection, males were collected for RNA-sequencing as well. All matings took place from morning to early afternoon. This was repeated on three different days, to collect three replicates for each treatment.

### Receptivity assay

We collected and mated Dad and control males and *Oregon-R-P2* virgin females as described above at 29 °C. After mating, males were discarded. Females were kept at room temperature for four days. Vials were checked for the presence of larvae to ensure the first mating had occurred, and females were presented with a virgin 3–5 day old *Oregon-R-P2* male. We recorded blinded if females remated within three hours at room temperature. We analyzed the remating data in R (R Core Team 2018) using a Fisher’s exact test.

Dissections, RNA extraction, library preparation and sequencing.

We dissected the female reproductive tract in sterile 1X PBS on a petri dish filled with dry ice (dry ice was used to keep the 1X PBS cool, but the flies were not frozen during dissections). The digestive system and the upper part of the common oviduct were removed. Reproductive tract samples were stored in groups of ten in 50 ul of Trizol and were mechanically homogenized using a pestle before being flash frozen and stored at -80 °C.

To extract RNA, we pooled all Trizol aliquots of the same sample and brought up the total volume to 1 ml. We extracted RNA using the Direct-zol RNA Microprep Kit according to the manufacturer’s instructions (Zymo, CA). We extracted RNA from on average 64 female reproductive tracts per sample. For male samples, we extracted RNA from three whole males. We measured the RNA concentration of all samples using Qubit, assessed purity using Nanodrop and verified the integrity of 12 out of 21 samples using the Agilent Fragment Analyzer at the Cornell Genomics Facility.

To sequence mRNAs and non-polyadenylated long noncoding RNAs, we used the Ovation Universal Drosophila RNA-Seq Kit, which allows for the depletion of abundant ribosomal RNAs (Tecan, CA). We prepared the libraries according to the manufacturer’s instructions (Tecan, CA). For the optional fragmentation step in the protocol, we fragmented cDNA using Covaris E220 to obtain a fragment size of 400 bases.

To sequence small RNAs, we used the NEBnext Small RNA library prep (New England Biolabs, MA), starting from 1 ug of total RNA (we used RNA from the same samples that were also used for RNA-seq; small RNA-seq was not done for male samples). We adjusted the NEB protocol at the following steps: 1) We ran the 3’ ligation reaction overnight at 16 °C to improve detection of any piRNAs that could be present in our samples. 2) We modified the protocol to remove abundant small rRNAs. To remove eight abundant small rRNA fragments, we used blocking oligos designed by Fowler et al. (2019) [39]. We incorporated these in the library preparation using a slightly modified method from Wickersheim et al. (2013) [96]. Specifically, we added 1 ul of an oligo cocktail (each oligo at a concentration of 10 uM) to the 3’ ligation reaction. We incubated this reaction for 2 min at 70 °C and for 5 min at 60 °C, before proceeding to the reverse transcriptase primer hybridization step. We purified the resulting libraries using the Monarch PCR & DNA cleanup kit (New England Biolabs, MA) and size selected using Pippin Prep (Sage Science, MA), selecting fragments of 120–250 bp. We tried these modifications first on test samples of RNA derived from whole virgin females. After sequencing at a depth of 10 M reads on Illumina’s NextSeq500 platform at the Cornell Genomics Facility, we verified that adding rRNA blocking oligos greatly reduced the number of rRNA reads in the final test library (Fig. S3).

All libraries were sequenced using single-end sequencing on the NextSeq500 platform at the Cornell Genomics Facility, where fastq files were generated using Illumina pipeline software v2.18.

### RNA-seq read processing and differential expression analysis

We investigated library quality using fastqc (https://www.bioinformatics.babraham.ac.uk/projects/fastqc/). We used Trimmomatic to remove overrepresented sequences and to filter reads [97]. Specifically, bases at the end of a read with a Phred quality score less than 20 were clipped off. The read was also clipped once, in a window of 5 bases, the average read quality dropped below a Phred score of 20. After trimming, we kept only reads with a minimum length of 30 bases. On average, libraries consisted of 14.4 M reads. Reads were aligned to dm6 using STAR [98] and read counts were obtained using HTSeq [99].

We performed differential expression analyses using edgeR [100]. A principal component analysis demonstrated that female reproductive tract samples clustered as expected (Fig. S4) and all replicates correlated well with each other based on a Pearson’s correlation (correlations ranged from 0.948–0.99). Reads were kept in the dataset if they had a cpm (counts per million) > 1 in at least three samples, keeping 9,863 genes in the dataset. We set up two pairwise contrasts to identify differentially expressed genes: 1) We contrasted females mated to a *w*^*1118*^ male with unmated females (all collected at room temperature); 2) We contrasted females mated to Dad males (*esg*^*ts*^* F/O UAS-Dad*) with females mated to a control male (*esg*^*ts*^* F/O w*^*1118*^) (all collected at 29 °C). Genes were called as differentially expressed based on a Benjamini–Hochberg adjusted *q*-value < 0.05 [101]. For this analysis 3 biological replicates were used per treatment.

When performing the differential expression analysis, some mated female samples showed a significant downregulation of ovary-specific genes. Since our samples consisted of the reproductive tract and lower oviduct only, this suggested that some virgin samples were contaminated with parts of the ovary. To take this into account, we identified 81 genes whose expression is biased to the ovary (based on FlyAtlas 2; [66]) and clustered samples based on their normalized expression (count per million) for these 81 genes (Fig. S5). Expression bias to the ovary was calculated for each gene as ovary(FPKM)/sum of female tissues(FPKM), which leads to a number between 0 and 1, with 1 indicating a stronger expression bias to the ovary relative to other tissues. The cutoff for ovary expression bias used here was 0.6. Similar clustering of samples was observed with an expression bias cutoff of 0.7. This method resulted in two clusters, one containing samples with higher expression of ovary-biased genes (U29_1, U29_2, U29_3, U21_11, MC29_1 and MC29_3) and one containing all other samples with lower expression of ovary-biased genes. We added an extra variable to the linear model in edgeR, assigning all samples to either one of these two clusters, to remove the effects of ovary-biased genes from the differential expression analysis.

For male samples, reads were kept in the dataset if they had a cpm (counts per million) > 1 in at least three samples, keeping 12,770 genes in the dataset. We set up a pairwise contrast between Dad males (*esg*^*ts*^* F/O UAS-Dad*) and control males (*esg*^*ts*^* F/O w*^*1118*^) with 3 biological replicates per treatment. Genes were called as differentially expressed based on a Benjamini–Hochberg adjusted *q*-value < 0.05 [101].

### MicroRNA-seq read processing and differential expression analysis

For the small RNA-seq libraries, we analyzed library quality using fastqc (https://www.bioinformatics.babrahamac.uk/projects/fastqc/), removed adapters using Cutadapt and kept reads with a length between 19 and 25 bp [102]. Reads were aligned to dm6 using bowtie2 [103], and featureCounts [104] was used to obtain counts for annotated microRNAs. Libraries contained on average 7.9 M reads.

Differential expression analysis was performed using edgeR [100]. A principal component analysis identified 3 outlier samples (MC29_3, MW21_1, U29_1), which we removed from the dataset (Fig. S6). This left us with 2 biological replicates for the MC29 (female mated to control male at 29 °C), MW21 (female mated to *w*^*1118*^ male at 21 °C) and U29 (unmated at 29 °C) treatments, and 3 biological replicates for the U21 treatment (unmated at 21 °C) and MD29 treatment (females mated to Dad male at 29 °C).

Reads were kept in the dataset if they had a cpm (counts per million) > 5 in at least 2 samples, keeping 164 microRNAs in the dataset. We set up the same contrasts as described above to identify differentially expressed microRNAs after mating to Dad or control males. We again added an extra variable to the linear model in edgeR to remove the effects of ovary-biased transcripts from the differential expression analysis. MicroRNAs were called as differentially expressed based on a Benjamini–Hochberg adjusted *q*-value < 0.05 [101]. We note that when running the analysis using all 21 samples (without outlier removal), no differentially expressed microRNAs were found in any contrast.

### GO term enrichment and figures

We used the R package ClusterProfiler [105, 106] for GO term enrichment analysis. We used all 9,863 genes expressed in our dataset as background, a minimum gene set size of 5, and a Benjamini–Hochberg correction for multiple testing [101]. All GO terms shown in this study had a q-value < 0.05. We consulted FlyBase to obtain additional information about genes of interest [107]. We used rrvgo [56] to reduce the number of significant GO terms based on related functionality. We used the R packages ComplexHeatmap [108], igraph [109] and ggplot2 [110] for data visualization.

## Supplementary Information

**Additional file 1. **Additional Tables

**Additional file 2. **Additional Results and Additional Figures

## Data Availability

Raw counts for the RNA- and microRNA-sequencing datasets are available on GEO (GSE216085).
