# Supplementary material for: Transcriptional programs are activated and microRNAs are repressed within minutes after mating in the Drosophila melanogaster female reproductive tract
Source: BMC Genomics. 2023 Jun 27;24:356. doi: 10.1186/s12864-023-09397-z (PMC10294459; doi:10.1186/s12864-023-09397-z)
Supplement: Supplementary file 2 — Additional file 2. Additional Results and Additional Figures [file 12864_2023_9397_MOESM2_ESM.docx]

# Additional Results

### Dad males have an altered transcriptional profile and induce different behavior in their mates relative to control males.

To ensure that all constructs used to generate Dad males were working as expected, we used RNA-seq to compare the whole-body transcriptome of Dad and control males, and we performed a receptivity assay on their mates, since earlier studies reported that Dad males are less able to induce refractory mating behavior in females (1,2). In a receptivity assay, 21/23 females that had previously mated with a Dad male remated with a wildtype male, 4 days after the first mating, while only 6/22 females that had previously mated with a control male remated (**Fig. S1A**; Fisher’s exact test *p-value* = 1.3*10^-5^).

Comparing the transcriptomes of whole Dad vs control males, we identified 92 differentially abundant transcripts, of which 75 underwent at least a 2-fold change in abundance (**Fig. S1B, Table S8**). Notably, we detected an almost 4-fold upregulation of *Dad* (log_2_ fold change = 1.98, *q-value* = 0.014) in Dad versus control males. The only significantly enriched GO term among the differentially expressed genes was “response to insecticide” (*Cyp12d1-p, Cyp6g1, Cyp6a8*). A manual search through the differentially expressed gene set, using FlyBase as a resource for gene information (3), yielded 15 long noncoding RNAs, of which one has a functional role in spermatogenesis (*lncRNA:CR43753*; (4)), and five genes with roles in cell division and DNA repair (*Blm, Mps1, ncd, phr, PolQ*). Differential expression of these genes could be explained by the fact that BMP signaling (which is inhibited by *Dad* overexpression) is required for the maintenance of stem cells (5). In addition, we identified differential expression of five genes that encode seminal fluid proteins (*Gld, CG9029, Spn75F, Acp24A4, CG13309*). A previous study reported differences in the proteomes of accessory glands of Dad and control males, but they did not detect significant differential abundance of any proteins encoded by these five genes (6). Overall, inhibition of BMP signaling in secondary cells, by overexpression of *Dad* using the *esg-GAL4* driver, led to only a moderate number of significant transcriptome differences between Dad and control males. It is possible that BMP signaling in secondary cells has a larger effect on protein-level changes rather than transcriptional changes, or more changes might be detected when RNA-seq is done on accessory glands only, rather than whole males. Still, the results from the receptivity assay and RNA-seq indicate that the overexpression of Dad and disruption of BMP signaling is effective. Thus, the lack of differential expression in the female’s response to mating with a Dad versus control male is unlikely to be caused by technical problems.

# Additional Figures


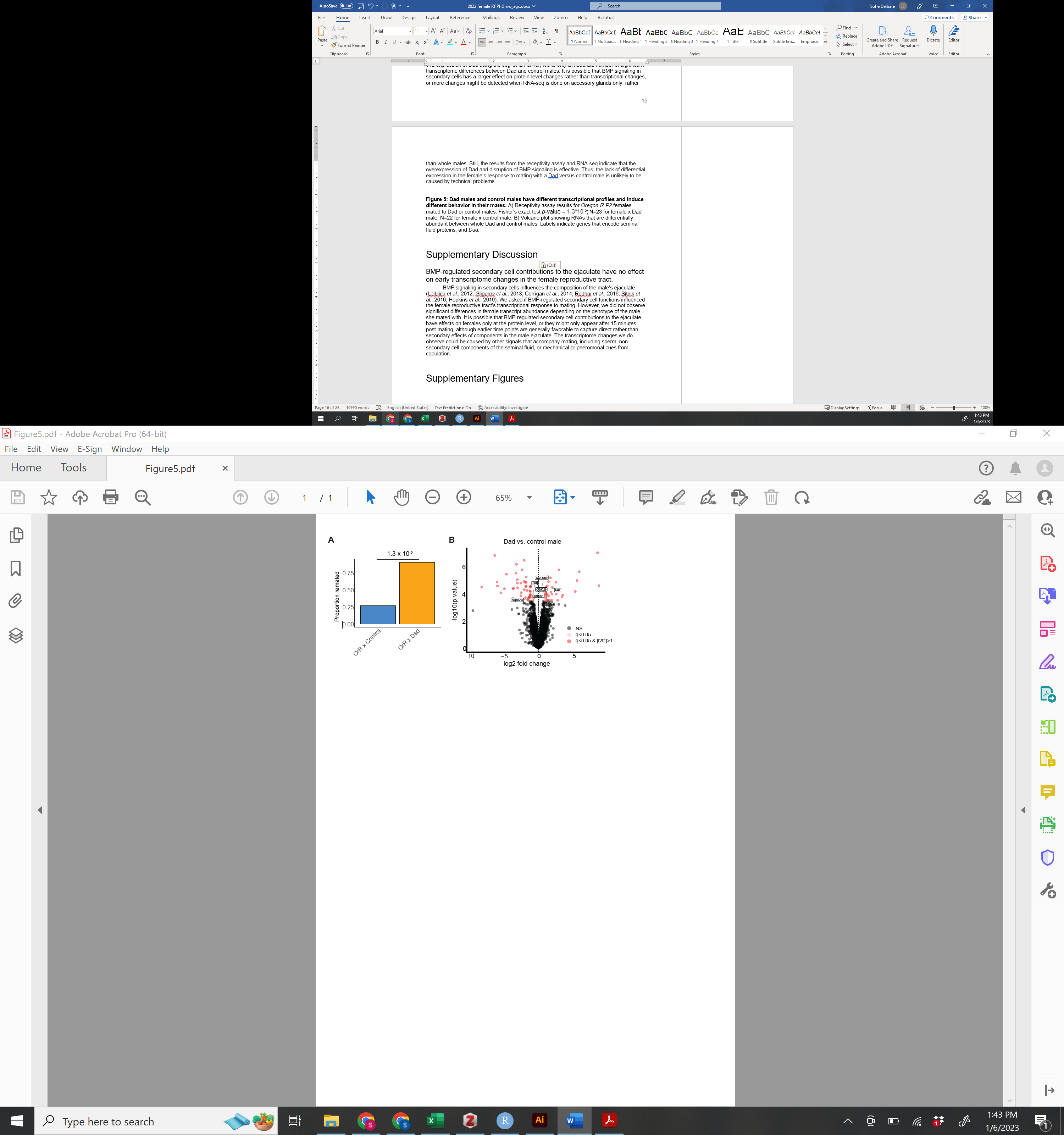


**Figure S1: Dad males and control males have different transcriptional profiles and induce different behavior in their mates.** A) Four-day receptivity assay results (shown as proportion remated) for *Oregon-R-P2* females. Females were first mated to either a Dad (OrR x Dad) or a control male (OrR x Control). Four days after this first mating, females were given three hours to remate with a *Oregon-R-P2* male to determine the remating proportion. Fisher’s exact test *p*-value = 1.3*10^-5^; N=23 for female x Dad male, N=22 for female x control male. B) Volcano plot showing whole-body RNAs that are differentially abundant between Dad and control males. Labels indicate genes that encode seminal fluid proteins, and *Dad*.

**Figure S2: Males might transfer RNAs to females during mating.** A) *Anp*; B) *Acp53C14b*. Coverage tracks from IGV are shown for males, and unmated and mated females (bam files were merged across replicates). Zoomed in figures for *Acp53C14b* show loci with very low expression in unmated females, and SNPs that differ from ones found in reads in males and mated females.


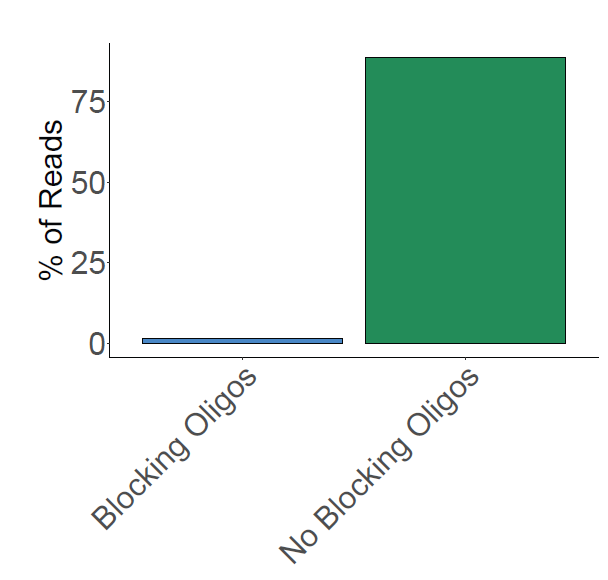


**Figure S3: rRNA blocking:** Percentage of reads aligning to ribosomal RNA in small RNA-seq libraries prepared with or without rRNA blocking oligos.


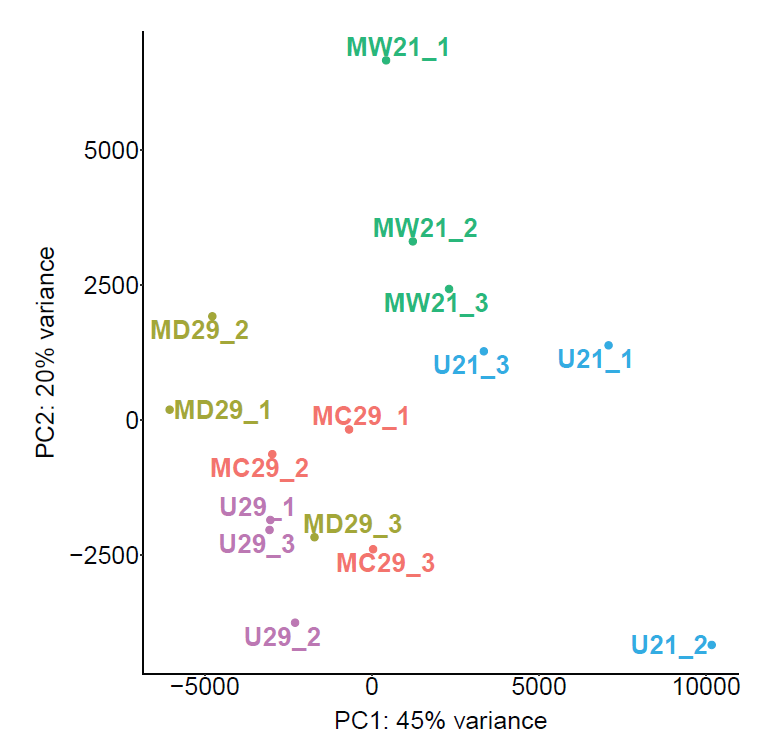


**Figure S4: Principal component plot for mated and unmated female RNA-seq samples.** MD = female mated to Dad male; MC = female mated to control male; MW = female mated to W^1118^ male; U = unmated female; Colors represent different treatments; 3 replicates were analyzed per treatment. 21 and 29 represent the temperatures (⁰C) at which matings occurred and flies were collected.


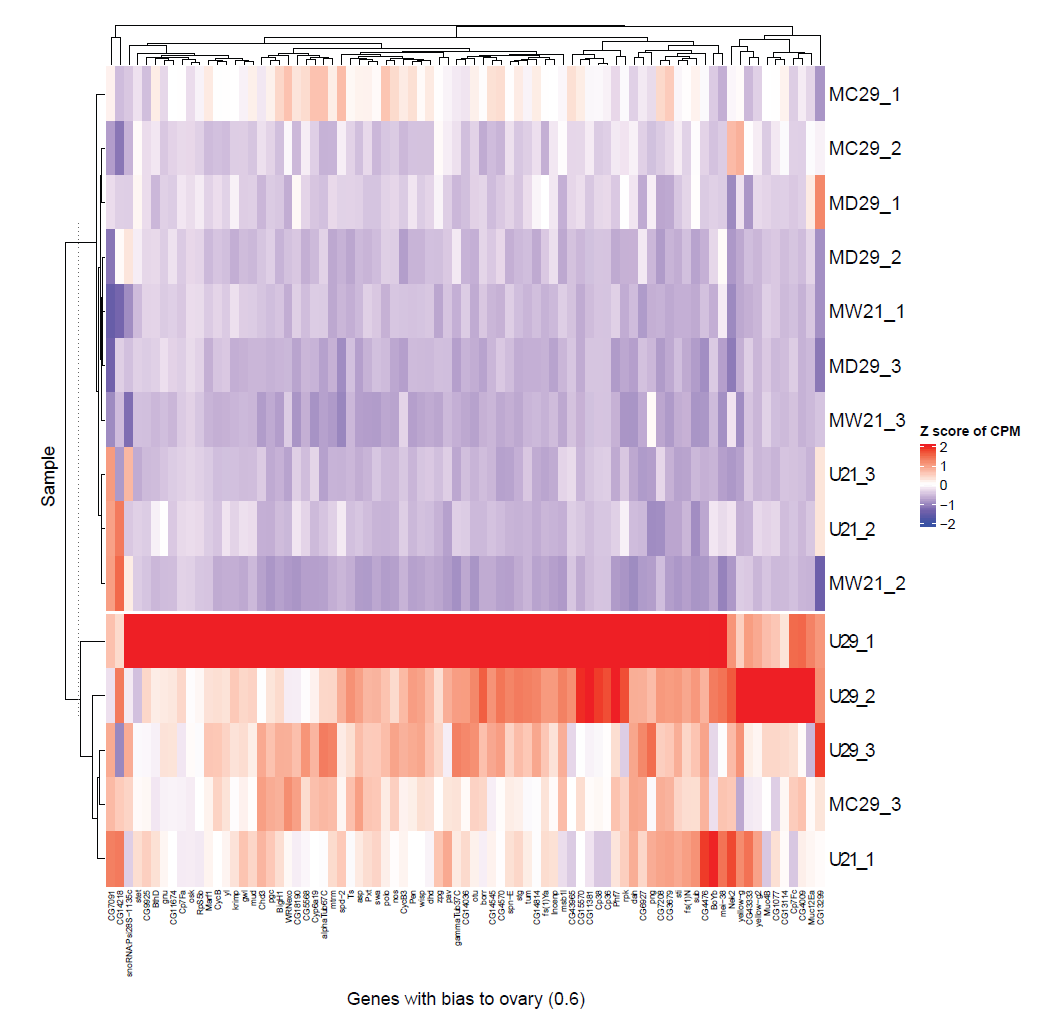


**Figure S5: Hierarchical clustering of female RNA-seq samples based on their CPM (count per million) for genes with an expression bias to the ovary.** Expression bias to the ovary was calculated for each gene as ovary(FPKM)/sum of female tissues(FPKM), which leads to a number between 0 and 1, with 1 indicating a stronger expression bias to the ovary relative to other tissues. Female-specific FPKM values were obtained from FlyAtlas2 (7). The cutoff for ovary expression bias used here was 0.6. Similar clustering of samples was observed with an expression bias cutoff of 0.7. MC = female mated to control male; MD = female mated to Dad male; MW = female mated to W^1118^ male; U = unmated female. 3 replicates were analyzed per treatment. 21 and 29 represent the temperatures (⁰C) at which matings occurred and flies were collected.


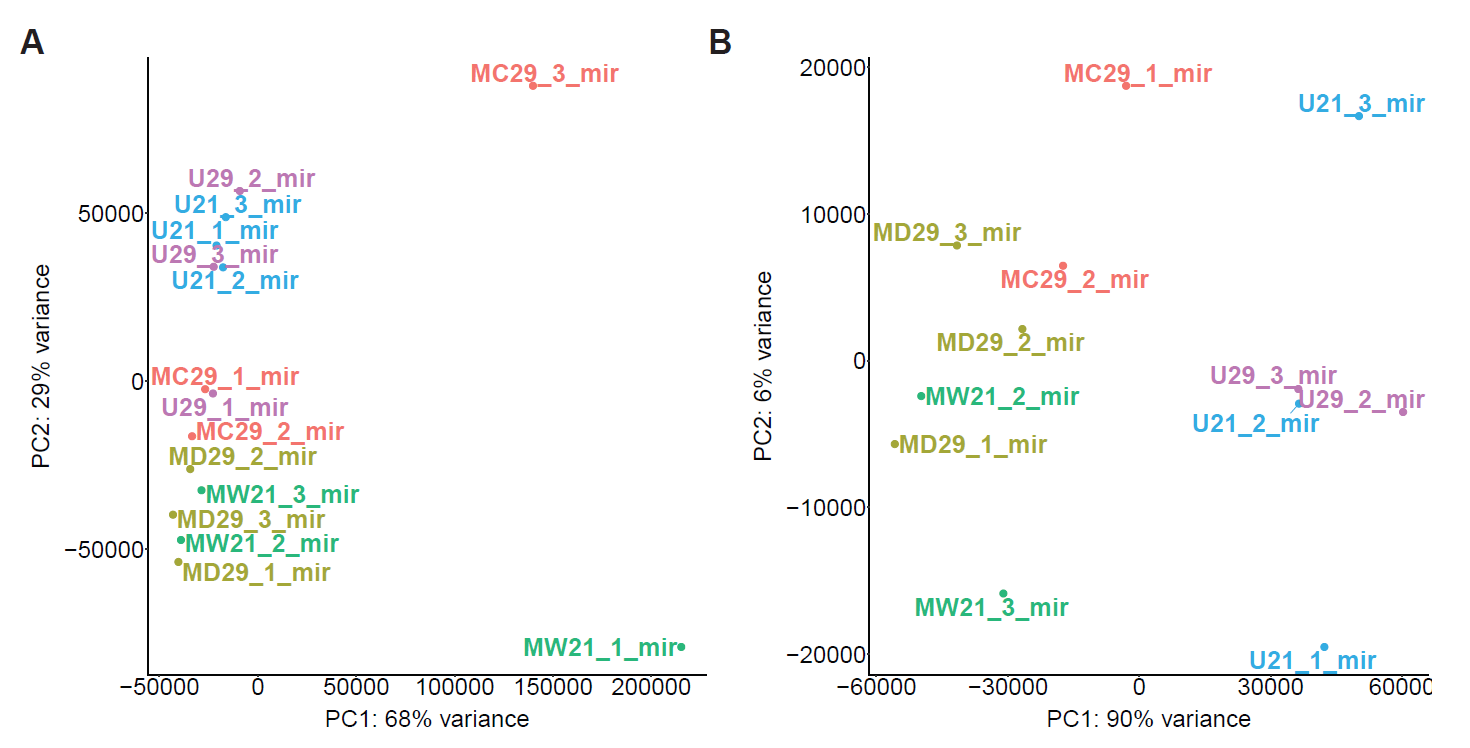


**Figure S6: Principal component plot for mated and unmated female microRNA-seq samples.** A) 3 samples appear as outliers in the plot (MC29_3, MW21_1 and U29_1, which localizes among mated female samples). B) Plot after removal of 3 outlier samples. MD = female mated to Dad male; MC = female mated to control male (genetic control for Dad males); MW = female mated to *w^1118^* male; U = unmated female; Colors represent different treatments; 3 replicates were analyzed per treatment. 21 and 29 represent the temperatures (⁰C) at which matings occurred and flies were collected.

# References

1. Leiblich A, Marsden L, Gandy C, Corrigan L, Jenkins R, Hamdy F, et al. Bone morphogenetic protein- and mating-dependent secretory cell growth and migration in the *Drosophila* accessory gland. Proc Natl Acad Sci. 2012;109(47):19292–7.

2. Corrigan L, Redhai S, Leiblich A, Fan SJ, Perera SMW, Patel R, et al. BMP-regulated exosomes from Drosophila male reproductive glands reprogram female behavior. J Cell Biol. 2014 Sep 1;206(5):671–88.

3. Jenkins VK, Larkin A, Thurmond J. Using FlyBase: A Database of Drosophila Genes and Genetics. In: Dahmann C, editor. Drosophila: Methods and Protocols [Internet]. New York, NY: Springer US; 2022. p. 1–34. Available from: https://doi.org/10.1007/978-1-0716-2541-5_1

4. Wen K, Yang L, Xiong T, Di C, Ma D, Wu M, et al. Critical roles of long noncoding RNAs in Drosophila spermatogenesis. Genome Res. 2016 Sep;26(9):1233–44.

5. Tian A, Jiang J. Intestinal epithelium-derived BMP controls stem cell self-renewal in *Drosophila* adult midgut. Banerjee U, editor. eLife. 2014 Mar;3:e01857.

6. Hopkins BR, Sepil I, Bonham S, Miller T, Charles PD, Fischer R, et al. BMP signaling inhibition in Drosophila secondary cells remodels the seminal proteome and self and rival ejaculate functions. Proc Natl Acad Sci. 2019 Dec 3;116(49):24719–28.

7. Leader DP, Krause SA, Pandit A, Davies SA, Dow JAT. FlyAtlas 2: a new version of the Drosophila melanogaster expression atlas with RNA-Seq, miRNA-Seq and sex-specific data. Nucleic Acids Res. 2018 Jan 4;46(D1):D809–15.
